# How Indoor Air Quality Affects Sleep in Older Adults: Evidence from the 2018 CLHLS

**DOI:** 10.1093/geroni/igaf122.247

**Published:** 2025-12-31

**Authors:** Cai Xu, Yen-Han Lee

**Affiliations:** Ohio University, El Paso, Texas, United States; University of Central Florida, Orlando, California, United States

## Abstract

**Background:**

Sleep is essential for health and well-being, particularly among older adults. This study examines the associations between indoor air quality and sleep outcomes in older Chinese adults.

**Method:**

We analyzed cross-sectional data from the 2018 Chinese Longitudinal Healthy Longevity Survey (CLHLS). Latent class analysis (LCA) identified ventilation patterns based on seasonal indoor air quality variations. Multiple linear and logistic regression models examined associations between ventilation patterns, air purifier use, home proximity to major roads, and sleep outcomes (quality and duration). Interaction effects between rural-urban residence and ventilation patterns were also assessed.

**Results:**

Among 9,902 adults (≥65 years), three ventilation classes were identified: Moderate with Summer-Selective Ventilation (11.2%), Strong Year-Round Ventilation (70.3%), and Minimal Ventilation (18.7%). Older adults with Strong Year-Round Ventilation (OR = 1.16, p < 0.01) and Moderate with Summer-Selective Ventilation (OR = 1.19, p < 0.05) had significantly better sleep quality. After accounting for interactions, urban residents with Strong Year-Round Ventilation had significantly higher odds of good sleep quality (OR = 1.27, p < 0.05). Additionally, older adults with Strong Year-Round Ventilation slept 0.30 hours longer per day (p < 0.001). After interactions, urban older adults with Moderate with Summer-Selective Ventilation slept 0.46 hours longer per day (p < 0.01).

**Conclusion:**

Indoor air quality, particularly ventilation patterns, is associated with sleep quality and duration among older adults in China. Improving indoor air quality may be an effective strategy to promote healthier sleep, particularly for older adults in urban settings.

